# Notices Concerning the Dengue

**Published:** 1828-10

**Authors:** 


### Art. V.—*Notices concerning the Dengue.*

### 1. Editorial Remarks.

The physicians of the middle and northern states have learned through the newspapers, that within the present year, a febrile disease, exhibiting some novel and peculiar symptoms, has been epidemic in the West Indies and in the Southern border of the United States. From the Spanish inhabitants of the former, it has received the appellation of *El Dengue* or *affectation*, for what reason does not clearly appear. The physicians of the region where it has prevailed have not yet bestowed upon it an appropriate name, and perhaps it is not, in reality, entitled to such a distinction, for it may be nothing more than a modification of the great autumnal endemic of the South.

No case of this malady has fallen under our observation, and, as far as we have heard, it has not extended north of the 34th degree of latitude; except on board of steam boats, ascending the Mississippi from New-Orleans.

When on a late visit to a neighbouring town, one of our medical friends mentioned to us a mistake relative to this disease, which deserves to be stated, to the end, that others may guard against a similar error. A gentlemen taken ill, was pronounced by his physician to labour under *Dengue*, and accordingly, treated to his manifest injury, with hot sudorifics, for eight or ten days. At the end of that time, our informant was called into consultation, and found it a well marked case of *inflammatory Rheumatism*, which was cured by copious bloodletting and the usual antiphlogistic means.

We have been favoured by a young medical gentleman of South Alabama, now in this city, with a memorandum concerning this malady, which is inserted below. He has himself experienced it, and witnessed it, repeatedly, in others. We also find in an eastern Journal, just received, a communication from a West India physician, which we shall subjoin, as being the fullest account we have seen.

2. *Memoranda relative to the Dengue or Spanish Fever, by* Dr. D. W. Vance, of Alabama.

This disease made its appearance in New-Orleans, in the month of June last, and spread rapidly in all directions. It attacked at an early period, the inhabitants of all crowded places, such as taverns, hotels, and steam boats. It affected persons of all ages and colours, and in some cases attacked the same individual a second time, with increased violence. Some observers have told me, that children and females are, in general, more severely afflicted than men; but of this I am not able to say any thing decisive; I have seen, however, that corpulent and robust persons have it more violently than the lean.

My observations on this malady, were chiefly made on board the steam boat General Pike, during a voyage from New-Orleans to Louisville, in the month of August. There were about one hundred persons in the boat, and nearly the whole, including myself, experienced the disease.

*Of its symptoms.* The onset of the complaint is often severe and distressing. It is attended with flushed face, violent headache, giddiness, soreness and aching of all the bones, dry and harsh skin, in some cases spasms, prostration of strength, and high fever, with a hard, frequent, and full pulse. These symptoms continue, with but little abatement or alteration, for 12, 24, or 36 hours, and in plethoric habits for 48, or longer; during which time, the debility of the patient is extreme, and the aching of the joints in the worst cases, almost as great as if they were luxated. The nervous system, in all instances, seems to be greatly and generally disturbed; and it frequently leaves the intellectual functions in a state of debility and confusion. It has been sometimes mistaken for ordinary bilious fever, and treated accordingly, with very bad effect.

*Of the cure.* The remedies found most successful in the Dengue, are all of a mild nature. The most efficient are lemonade, herb teas, demulcents, and other diaphoretics; with a gentle cathartic. When spasms occur, laudanum, ether, and other diffusible stimuli are required. External applications have done good in some cases. Brandy and a liniment composed of burnt brandy simmered with olive oil or spermaceti, with frictions, are the means which I have seen employed externally. The warm bath has also been useful, especially when there was a tendency to spasms. Very different, however, is the effect of the cold bath, which as far as I know, is highly prejudicial. I have, indeed, seen it prove fatal in several instances. The lancet has been sometimes resorted to, but with little or no advantage.

When the Dengue recedes, the patient is left for some time with a considerable soreness of the limbs, which, in many cases, resembles chronic rheumatism. Of its effect on the mind I have already spoken.

It is highly necessary for every convalescent from this malady, carefully to guard against taking cold.

Such are the observations which I have had an opportunity of making, on this singular malady.

D. W. VANCE.

*Cincinnati, October,* 1828.

3. *Remarks on the Dengue.* By David Osgood, M. D. of Havana.

[From the Boston Medical and Surgical Journal, No. 36.]

Late in April, the present year, after a continuation of hot weather, with but slight variations, during the different seasons of this and the last preceding year, (1827 and 1828,) a new epidemic fever arose in Havana and its vicinity, which attacked the inhabitants generally, and almost simultaneously.

The same disease took place, earlier in the spring, in the other seaport towns and cities of the West Indies; and in the course of the summer it has appeared in some of those of the United States.

The people of Havana have named this strange fever *El Dengue*, which word signifies, literally, affectation.

During the whole of the last winter and spring, previously to the taking place of this new fever, many of the persons who had lately arrived in this climate were seized with the yellow fever. But as soon as the former began to prevail, the latter disappeared; although the residents, who had usually been exempt from the yellow fever, were seen, as well as the transient subjects, with symptoms resembling those of this latter disease, viz: after sensations of uncommon languor, chilliness, and pain in the tendons of the smaller joints, they were suddenly attacked with a burning heat and redness of the skin, pains in the muscles of the limbs, or pain in the forehead, and a loathing, or vomiting of whatever was taken into the stomach.

The fever continued for one, two, or three days, and then usually terminated with a free sweating, which freed the patient likewise from his pains. But many after leaving their beds suffered by a renewal of their pains, which in some have become chronic. Others have also had a renewed attack of the fever.

This disease of the season has not proved fatal, except to a few amongst the strangers, in whom the sweating stage of it did not easily take place, or was suddenly stopped by exposure to the open air.

This moderation of the symptoms, in the generality of the subjects, I attribute to a gradual reduction of the vigor of their constitutions, by the influence upon them of the before-mentioned almost uninterrupted continuance of hot weather during all the seasons of the two last years past, and beyond what has happened in former years; which influence has rendered the native inhabitants, as well as the strangers in the climate, liable to be affected in this new way, by the same specific cause that, at other times, has produced the yellow fever in the latter class of the subjects.

The Dengue has, as yet, only prevailed in the places to which the yellow fever has been limited. It has not spread into the interior of Cuba, although at the end of five months from the time ofits rise in Havana, it continues to attack most of the persons who come to the city from the country, or from any place where it has not prevailed.

2. The fatality of the yellow fever is known to be the greatest in the most robust of its subjects; and in those who, besides their recent exposure to the air of the West India climate, indulge themselves in hard drinking, or fatigue themselves by much exercise in the sun, whereby their strength is *suddenly depressed.*

3. On the contrary, with a slight typhus infection contracted in a close prison, where hard drinking, or taking long walks, cannot be practised, the additional influence of the air of the climate, when it has caused a fever by sudden exposure to it, the symptoms have generally been very mild.

4. The late comers to the West Indies from the colder latitudes, experience, soon after their arrival, a degree of exhaustion, and soreness in their muscles, like that which persons every where feel after violent exercise, or very great fatigue.

The debility and soreness of the muscles and tendons in the subjects with the dengue, before and after their symptoms of fever, may be attributed to the same cause.

5. In several instances, since the prevalence of the dengue, the worst symptoms of the yellow fever have supervened on those of the former.

I may also mention, here, that I know several persons now long residents in this place, who since arriving here from abroad have escaped from an attack of the yellow fever, and have not hitherto been attacked by the dengue; whereas every other person to whom I put the question, "Have you had the dengue?" answers "Yes."

From the facts, &c. above stated, I have been led to consider the specific cause of the disease of the present time, and that of the yellow fever, to be the same. The subjects have become altered in their constitutions; but the generating cause both of the new and of the old fever, remains unchanged.

Although aware that this opinion may be thought too theoretical, yet I venture to advance it for the sake of a practical inference to be drawn from the viewing of it in this light.

In the dengue, as before noticed, when a sweating takes place, it carries off the burning heat and redness of the skin, and at the same time the loathing and sickness of the stomach. Hence, for the most part, no other than the antiphlogistic treatment has been found beneficial to the patients; but for the few in whom, under such treatment, a sweat has not easily flowed by the end of the second, or the beginning of the third day, the more active, and especially depleting means as in the yellow fever, have been found requisite.

But as the black vomit, the most dreaded symptom of the yellow fever, cannot, with certainty, be overcome by any remedy received into the stomach, to act directly upon that organ, we may take a hint from the dengue, and regard the affection of the stomach as being sympathetic with that of the skin, in its burning state. Agreeably to this idea, it is well known, that, by means of tepid emollient fomentations and clysters, and such diluting drinks as are best calculated to remove thirst, perseveringly employed early in the disease, and at a later period of it by more stimulating applications to the skin, the black vomit has often been prevented, and sometimes stopped

